# Removal of Mixed-Species Biofilms Developed on Food Contact Surfaces with a Mixture of Enzymes and Chemical Agents

**DOI:** 10.3390/antibiotics10080931

**Published:** 2021-07-30

**Authors:** Maricarmen Iñiguez-Moreno, Melesio Gutiérrez-Lomelí, María Guadalupe Avila-Novoa

**Affiliations:** 1 Centro de Investigación en Biotecnología Microbiana y Alimentaria, Departamento de Ciencias Básicas, División de Desarrollo Biotecnológico, Centro Universitario de la Ciénega, Universidad de Guadalajara, Av. Universidad 1115, Col. Lindavista, 47820 Ocotlán, Jalisco, Mexico; mari.moreno2312@gmail.com; 2 Laboratorio Integral de Investigación en Alimentos, Tecnológico Nacional de México/Instituto Tecnológico de Tepic, Av. Tecnológico 2595, 63175 Tepic, Nayarit, Mexico

**Keywords:** stainless steel, polypropylene, organic matter, microbial resistance, peracetic acid

## Abstract

Sanicip Bio Control (SBC) is a novel product developed in Mexico for biofilms’ removal. The aims of this study were to evaluate (i) the removal of mixed-species biofilms by enzymatic (protease and α-amylase, 180 MWU/g) and chemical treatments (30 mL/L SBC, and 200 mg/L peracetic acid, PAA) and (ii) their effectiveness against planktonic cells. Mixed-species biofilms were developed on stainless steel (SS) and polypropylene B (PP) in whole milk (WM), tryptic soy broth (TSB) with meat extract (TSB+ME), and TSB with chicken egg yolk (TSB+EY) to simulate the food processing environment. On SS, all biofilms were removed after treatments, except the enzymatic treatment that only reduced 1–2 log_10_ CFU/cm^2^, whereas on PP, the reductions ranged between 0.59 and 5.21 log_10_ CFU/cm^2^, being the biofilms developed in TSB+EY being resistant to the cleaning and disinfecting process. Higher reductions in microbial load on PP were reached using enzymes, SBC, and PAA. The employed planktonic cells were markedly more sensitive to PAA and SBC than were the sessile cells. In conclusion, biofilm removal from SS can be achieved with SBC, enzymes, or PAA. It is important to note that the biofilm removal was strongly affected by the food contact surfaces (FCSs) and surrounding media.

## 1. Introduction

Biofilms are growing communities of microorganisms adhered to a surface and were embedded in self-produced extracellular polymeric substances (EPS) [1]. The type and amount of EPS are strain-dependent and vary with the environmental conditions in which biofilms are formed. Nevertheless, the general composition of EPS includes polysaccharides, proteins, lipids, and extracellular DNA [1,2]. Biofilm development confers advantages to microbial cells, such as physical resistance to refrigeration, heat, desiccation, acidity, and salinity; mechanical resistance to liquid streams in pipelines; and chemical protection against antimicrobials and disinfectants [3,4]. Otherwise, biofilms cause corrosion in equipment, biofouling in water systems, and post-process contamination contributing to food spoilage. Recently, biofilms have been associated with the generation of foodborne diseases [5].

Almost 800 foodborne disease outbreaks are reported every year in the USA, causing approximately 15,000 foodborne illnesses, 800 hospitalizations, and 20 deaths [6]. The National Institutes of Health estimated that over 65% of microbial diseases are related to biofilm formation [7]. *Listeria monocytogenes*, *Salmonella*, and Shiga toxin-producing *Escherichia coli* are related to 82% of all hospitalizations and deaths in the USA. Another important biofilm-former microorganism commonly implicated in foodborne diseases is *Bacillus cereus* (2%) [6].

In the last decade, food industries have focused on managing food spoilage caused by biofilm-forming microorganisms, such as *Clostridium*, *Brochothrix thermosphacta*, Enterobacteriaceae, lactic acid bacteria, *Pseudomonas* spp., and *Bacillus* spp. [8,9]. The Food and Agricultural Organization of the United Nations [10] reported that over 25% of global food production is lost by microbial action (bacteria, yeasts, and molds). These microorganisms have been associated with biofilm development in dairy, meat, and egg processing industries [9].

Sanitization programs are the main alternative to biofilm control in the food industry [11], which comprise two phases: cleaning and disinfection [5]. The cleaning process removes food residues (proteins, fats, minerals deposits, sugars, and others) and 90% of the microorganisms from food contact surfaces (FCSs) [12]. Disinfection is the application of physical methods (UV light, cold plasma, ultrasound, etc.) or chemical agents (biocides and antimicrobials) to cause damage to or kill microorganisms [13]. Nonetheless, the use of physical [14] or chemical methods [15,16] is not enough to remove and eradicate the microorganisms within the biofilm, because the EPS occludes them with antimicrobial agents, reducing the shear forces [5,17]. Moreover, disinfectants cannot remove the biofilms; therefore, non-removed biofilm modify the surface charge and serve as a new substrate to other microorganisms, enabling them to restart the biofilm formation again [15]. Therefore, it is necessary to consider the introduction of different strategies, such as the use of enzymes, to achieve the biofilm removal [18]. Enzymes can kill bacteria and break down the biofilm structure due to EPS disruption [5,9]. CIP & GROUP is a Mexican company that recently developed Sanicip Bio Control (SBC). This is a cleaner and disinfectant product based on a mixture of high penetration surfactants, quaternary ammonium compounds of fifth-generation, and oxidant agents. The purpose of SBC is to achieve the biofilm removal from FCSs and kill the microorganisms that developed it.

Previous research demonstrated that *Salmonella*, *E. coli*, *L. monocytogenes*, *B. cereus*, and *Pseudomonas aeruginosa* developed mixed-species biofilms onto stainless steel (SS) and polypropylene B (PP), in whole milk (WM), and in culture media with egg yolk or meat extract [19]. In the food industry, biofilms are composed of multiple microorganisms; therefore, it is important to evaluate the cleaning and disinfection process in mixed-species biofilms developed under conditions that simulate food processing environments. Therefore, this study aimed to evaluate (i) the effect of enzymatic and chemical treatments on biofilm removal, (ii) examine the addition of peracetic acid to the disinfection process, and (iii) compare the effect of chemical agents against planktonic cells and mixed-species biofilms developed in the presence of organic matter or food residues.

## 2. Results

### 2.1. Microbicidal Activity against Planktonic Cells

**Figure 1:**
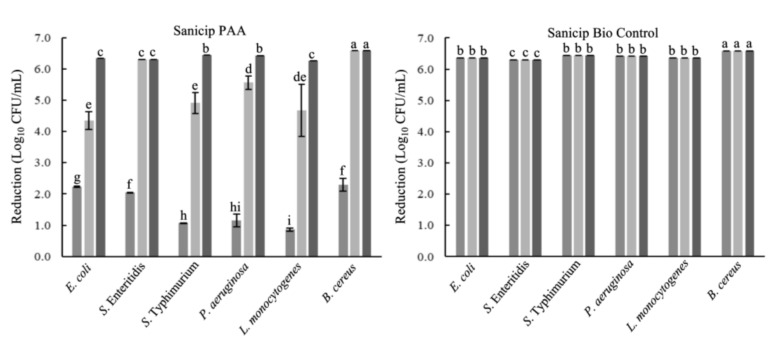
Microbicidal activity against planktonic cells in the presence of organic matter. Each bar represents the mean of three tests of the antimicrobial activity ± standard deviation. Sanicip PAA (200 mg/L) or Sanicip Bio Control (8 mL/L) in meat extract (■; 100 g/L); egg yolk (■; 100 mL/L); and whole milk (■; 100 mL/L). Bars within the same graph with different lower-case letter are significantly different according to Fisher’s LSD test at *p* ≤ 0.05.

*E. coli*, *Salmonella* Typhimurium, *Salmonella* Enteritidis, *P. aeruginosa, L. monocytogenes*, and *B. cereus* were reduced by 99.9999% (>6 log_10_ CFU/mL) after 30 s of contact with peracetic acid (PAA) or SBC, in suspensions without food residues. In the presence of food residues, SBC maintains its efficacy (99.9999%). However, the activity of PAA decreased in egg yolk and meat extract (p ≤ 0.05) in comparison to that in WM. PAA reduced ~4 log_10_ CFU/mL of *E. coli*, *S.* Typhimurium, and *L. monocytogenes* in egg yolk; otherwise, in meat extract the reductions ranged from 0.86 to 2.30 log_10_ CFU/mL (Figure 1).

### 2.2. Biofilm Removal on SS

**Figure 2:**
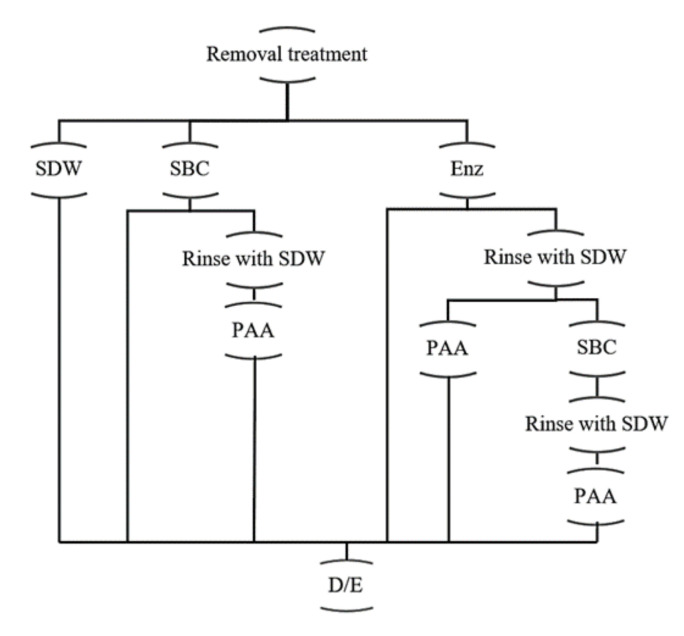
Treatments applied to removal and disinfection of mixed-species biofilms. SDW: sterile distilled water; SBC: Sanicip Bio Control (30 mL/L, 30 min, 25 °C); PAA: Sanicip PAA (200 mg/L, 10 min, 25 °C); Enz: enzymatic treatment (180 MWU/g, 30 min, 25 °C; MWU: modified Wohlgemuth unit); D/E: Dey/Engley broth (3 mL, 30 min).

**Figure 3:**
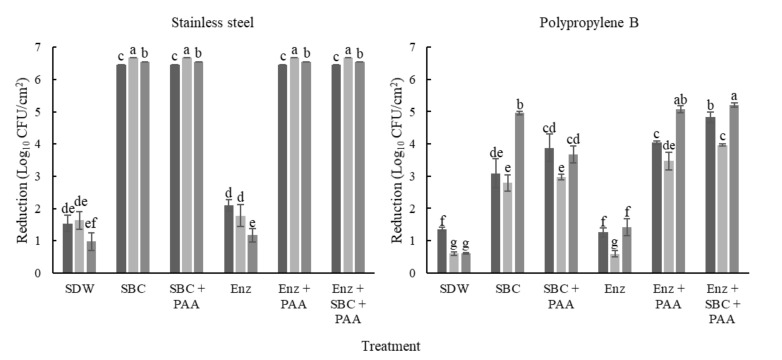
Reductions in mixed-species biofilms after removal and disinfection treatments. The biofilms were developed on stainless steel (SS) and polypropylene B (PP), in TSB with 100 g/L meat extract (■), TSB with 100 mL/L egg yolk (■), and whole milk (■) and were incubated at 25 °C for 240 h. Each bar represents the mean of three tests ± standard deviation of the means (*n* = 3) of cell density after removal treatments; SDW: sterile distilled water; SBC: Sanicip Bio Control (30 g/L, 30 min, 25 °C); PAA: Sanicip PAA (200 mg/L, 10 min, 25 °C); Enz: enzymatic treatment (180 MWU/g, 30 min, 25 °C; MWU: modified Wohlgemuth unit). Bars within the same graph with different lower-case letter are significantly different according to Fisher’s LSD test at *p* ≤ 0.05. Detection limits were 0.71 and 0.81 log_10_ CFU/cm^2^ for PP and SS, respectively.

To evaluate biofilm removal, mixed-species biofilms were developed on SS and PP coupons in three culture media. A mixture of proteolytic and amylolytic enzymes, and SBC were used for biofilm removal, and PAA was applied as a disinfectant (Figure 2). In biofilms developed on SS, the cellular densities ranged from 6.46 to 6.67 log_10_ (Table 1). Moreover, we analyzed the population before and after the treatments. In this regard, differences in the initial count between each species were observed (*p* ≤ 0.05; Table 1). In addition, all treatments (except the enzymatic) reached over 6 log_10_ CFU/cm^2^ of microorganism reduction in the mixed-species biofilms on SS. After enzymatic treatments, the counts of biofilms developed in tryptic soy broth (TSB) with 100 mL/L chicken egg yolk (TSB+EY) and whole milk (WM) were not different to those of their control (*p* > 0.05; Figure 3). However, the cell density of P. aeruginosa in biofilms developed in TSB+EY was reduced after the enzymatic treatment (*p* ≤ 0.05). The same occurred with *E. coli* and *L. monocytogenes* in WM (*p* ≤ 0.05, Table 1).

### 2.3. Biofilm Removal on PP

The initial biofilm counts were higher on PP (~7.49 log_10_ CFU/cm^2^) than on SS (*p* ≤ 0.05). Furthermore, the biofilms showed greater resistance on PP compared to on SS, with reductions between 0.59 and 5.21 log_10_ CFU/cm^2^ (*p* ≤ 0.05; Figure 3). However, significant differences were observed in the initial counts of each microorganism (*p* ≤ 0.05). On PP, the low reductions were obtained with enzymatic treatments (*p* > 0.05, Figure 3). Moreover, E. coli and L. monocytogenes were recovered of biofilms developed in TSB with meat extract (TSB+ME) after treatments with SBC with or without the previous enzymes’ application (Table 2).

Even when the initial counts of Salmonella and P. aeruginosa were similar (*p* > 0.05) in biofilms developed in TSB+ME and TSB+EY; Salmonella loads were higher than those of P. aeruginosa after all treatments (*p* ≤ 0.05). Moreover, these microorganisms were recovered after all treatments applied on PP, with cellular densities between 2.37 and 4.63 Log_10_ CFU/cm^2^. 

In general, the microorganisms in the biofilms developed on PP were more resistant to removal and disinfection treatments than in the other residues or onto SS. L. monocytogenes were not recovered on biofilms developed in WM after treatments with PAA. Moreover, B. cereus was not quantified before treatments in biofilms developed in WM, and in most cases, was fully reduced after the removal and disinfection process (Table 2).

### 2.4. Epifluorescent Microscopy and SEM Analyses

**Figure 4:**
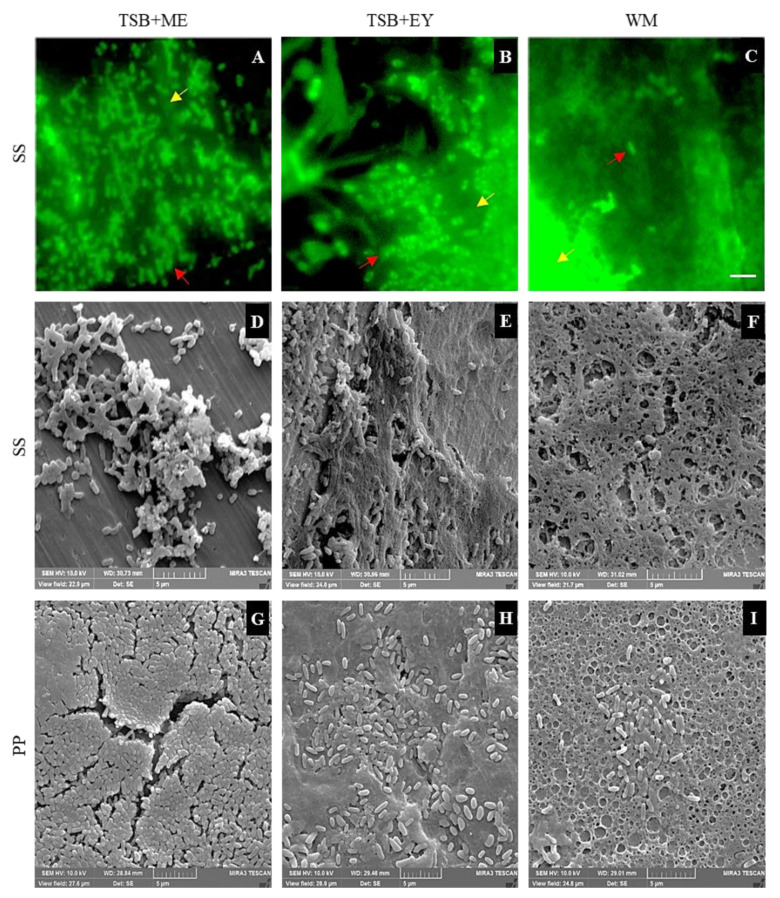
Micrographs of mixed-species biofilms. The micrographs were obtained by epifluorescence microscopy (**A**–**C**) and SEM (**D**–**I**) after 240 h of incubation at 25 °C of mixed-species biofilms in TSB with 100 g/L meat extract (TSB+ME), TSB with 100 mL/L egg yolk (TSB+EY), and whole milk (WM). The biofilms were developed on stainless-steel (SS) and polypropylene B (PP). The white bar scale indicates 5 μm. The red arrows shown the presence of metabolically active cells, whereas the yellow arrows indicate the presence of EPS and food residues.

**Figure 5:**
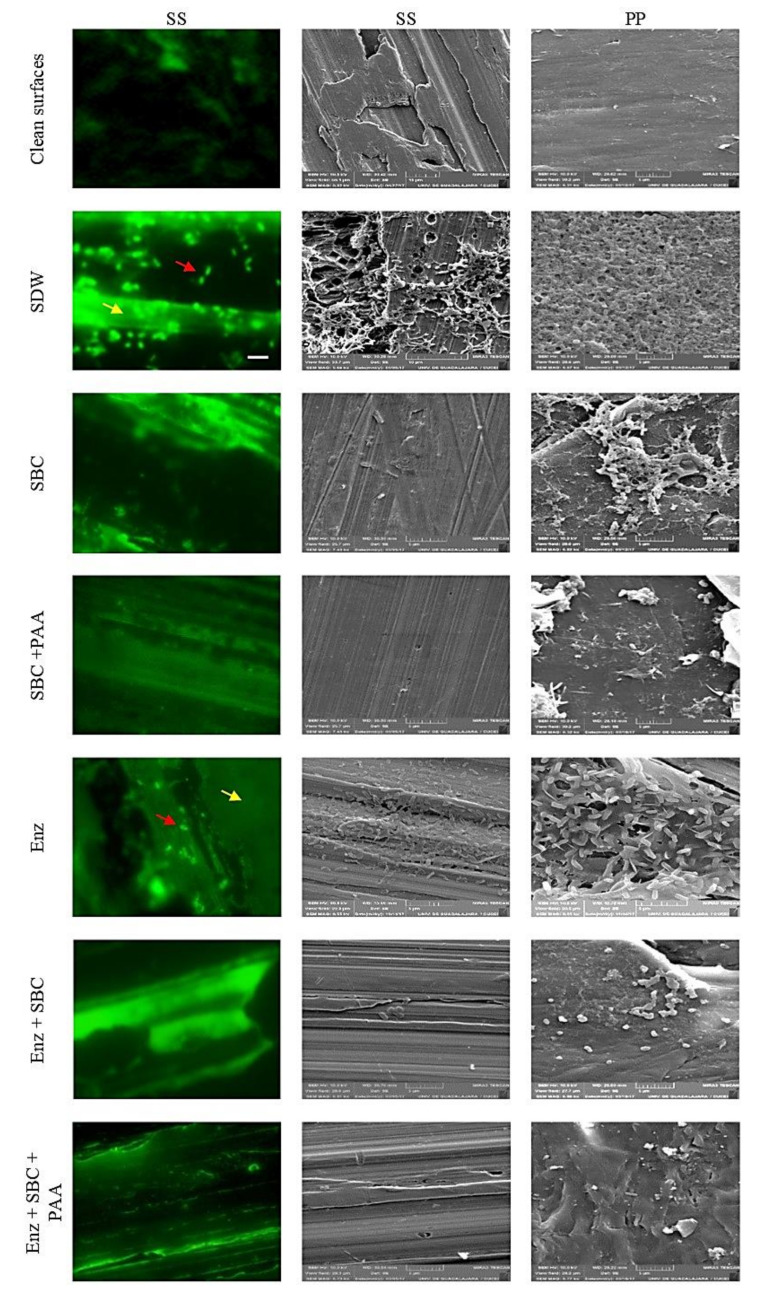
Micrographs of mixed-species biofilms after removal treatments. The mixed-species biofilms were developed on stainless steel (SS) and polypropylene B (PP) in whole milk during 240 h. The micrographs were obtained by epifluorescence microscopy and SEM after removal treatments SDW: sterile distilled water; SBC: Sanicip Bio Control (30 g/L, 30 min, 25 °C); PAA: Sanicip PAA (200 mg/L, 10 min, 25 °C); Enz: enzymatic treatment (180 MWU/g, 30 min, 25 °C; MWU: modified Wohlgemuth unit). The white bar scale indicates 5 μm. The red arrows shown the presence of metabolically active cells, whereas the yellow arrows indicate the presence of EPS and food residues.

Representative micrographs of mixed-species biofilms developed on SS in the different culture media were obtained by SEM and epifluorescence microscopy (Figure 4). In concordance with the counting plate technique before removal treatments, metabolically active cells were observed by epifluorescent microscopy. Furthermore, EPS and food residues were observed (Figure 4A–C) and confirmed by SEM (Figure 4D–I). After the removal and disinfection process of biofilms developed on SS, metabolically active cells were not observed by epifluorescence microscopy, except on the coupons with enzymatic treatment. However, through SEM, some bacterial cells and residues of EPS were observed, particularly after treatments with enzymes (Figure 5). Otherwise, cells and EPS were detected on PP after all treatments. Nevertheless, the biofilms were considerably removed in comparison to the images obtained before the treatments (Figure 4). After enzymes use, the microorganisms were easily observed due to EPS removal (Figure 5).

## 3. Discussion

Biofilms contribute to pathogen spread and food contamination, cause damage to food processing equipment, and increase antimicrobial resistance, representing significant losses to the public and private sectors [5,20]. In this study, we assessed the effect of different treatments against planktonic and mixed-species biofilms developed under conditions that simulate a food processing environment. The assessed products reached reductions of >5 log_10_ CFU/mL against planktonic cells. A reduction of 5 log_10_ CFU/mL is the minimum to consider a disinfectant as effective [21,22]. An inappropriate cleaning process can leave up to 100 g/L of organic matter [23]; hence, microbicidal tests were also carried out in the presence of food residues. The efficacy of PAA was reduced in egg yolk and meat extract due to the fact that proteins and fats affect the availability of the oxidant agents [24]. Considering the time exposition used in this research (30 s), the reduction obtained (99.99%) was higher than that in other studies. For example, the products Suma Tab D4 and Suma Bac D10 (quaternary ammonium compounds, 240 and 740 mg/L, respectively) reduced 5 log_10_ of *L. monocytogenes* after 5 min in whole milk [23].

Biofilms are the main bacterial lifestyle in food processing environments, and sessile microorganisms are more resistant than are planktonic cells. Therefore, we evaluated the effect of different treatments on the removal of mixed-species biofilms developed in different culture media (tryptic soy broth (TSB) with chicken egg yolk (TSB+EY), and with meat extract (TSB+ME), and WM). SDW treatments (included as controls), showed significant reductions in *P. aeruginosa*, *S. enteritidis*, and *S. typhimurium*. SDW can cause cellular lysis, due to solutes absence, and it can also dissolve simple sugars, mineral salts [25], and some cellulose structures [26]. The use of chemical agents for biofilm control in food environments is not always effective; therefore, their efficacy should be improved by the combination of biological agents and physical methods. 

Enzymes represent a great alternative for biofilm removal [27]. Biofilms in the food processing environment are composed of multiple microorganisms, resulting in an EPS with a heterogenic composition [28]. Recently, it has been demonstrated that polysaccharides in biofilms developed by Gram-negative bacteria, such as alginic acid, are the main component of the EPS matrix [13]. For example, the EPS matrix of *S.* Typhimurium is mainly composed of aggregative fimbriae and extracellular polysaccharides (cellulose) [29]. In contrast, proteins are the main compound in biofilms of Gram-positive bacteria [3]; however, they also produce polysaccharides as well as dextran [30]. Therefore, it is recommended to use a mixture of enzymes, because these molecules have specific activity [31]. In this study, the removal of mixed-species biofilms was evaluated using a mixture of alkaline protease and α-amylase. On SS surfaces, biofilm removal ranged between 93.4 and 96.3%. The low removal of biofilms developed in TSB+EY on PP (12.2%) was attributed to the high content of lipids in the egg yolk, which were not decomposed by the enzymes applied [32]. Ripolles-Avila et al. [3] achieved a removal of ~2.3 log CFU/cm^2^ of *S*. Typhimurium on SS 304 with a mix of enzymes (protease, lipase, and amylase), which is in agreement with the findings in this study.

Kumari and Sarkar [33] used a serine protease, resulting in a complete reduction in *B. cereus* biofilms (4.08 log_10_ CFU/cm^2^) developed in skim milk. The difference between this report and our results could be explained by the low cell density in the biofilms; moreover, after 24 h of incubation, the EPS matrix is not mature [19,34]. The EPS matrix is an important component of biofilms and represents more than 90% of the total mass of these structures [35]. The EPS matrix is the first resistance mechanism of the microorganisms in the biofilms against chemical and physical agents and environmental conditions. EPS components can react with the disinfectant molecules, protecting the microorganisms in the biofilm [36]. Recently, it has been reported that the application of an enzymatic cleaner (1 h at 50 °C) reduced 79.72% of *S. enterica* biofilm [28]. Nonetheless, to achieve this reduction, the samples were exposed for a least 1 h at 50 °C; this procedure is not viable for real conditions on an open surface in the food industry, a fact that was not considered in that report. Our study, however, was designed considering the application of the removal process on open surfaces in that environment.

To improve biofilm removal, enzymatic and chemical treatments were applied. With the combination of these treatments, microorganisms were not recovered from biofilms developed on SS. On PP, the reductions with the enzymatic and chemical treatments ranged from 3.06 to 4.76 log_10_ CFU/cm^2^ (Figure 3), and these results were greater than those reported in other studies [36]. The selection of the type of FCS is vital in the food processing environment. The high biofilm removal on SS is related to its hydrophilic nature, the presence of metallic ions on the surface [37], the germicidal activity of the quaternary ammonium compounds, and the organic acids in the SCB. In previous research, it was demonstrated that PAA at 3500 mg/L kills the cells in biofilms of *Staphylococcus aureus* without removing them [15]. Moreover, lower reductions were obtained on PP, because hydrophobic surfaces increase cell aggregation and biofilm development [15,19]. The aqueous solution has minimal contact with the surface of PP; even SBC has quaternary ammonium compounds and surfactants that decrease the superficial tension of water, facilitating biofilm removal [27]. These compounds and the organic acids in their formulation promoted biofilm removal from SS.

*Salmonella* and *P. aeruginosa* were recovered in great amounts after the removal treatments. This is related to their high counts before the treatment; moreover, it has been demonstrated that the biofilm formation by *Salmonella* is favored in the presence of other bacteria such as *Pseudomonas* sp. and *Bacillus* sp. [38]. In addition, it was reported that *Salmonella* biofilms were more sensitive to disinfectants when they were developed on SS than on PP [15]. Microorganism aggregation within a three-dimensional structure can provide protection against biocides activity [39]. Almeida et al. [39] observed that two well-defined layers exist in tri-species biofilms, on the surface of *E. coli* and in the deep mixed regions of *L. monocytogenes* and *S. enterica*. This can explain the absence of *E. coli* after the application of all treatments. 

Some studies have reported a higher resistance in sessile than in planktonic microorganisms to antimicrobials [40,41]. In line with this, the PAA reduced 2–3 log_10_ CFU/mL more in the assays with planktonic cells than in biofilms developed on PP treated with SBC or enzymes and then PAA. In addition to the EPS matrix, the presence of catalase in the microorganisms could play a role in peracetic acid decomposition. Unfortunately, the resistance mechanisms involved in mixed-species biofilms are not entirely clear [42].

Biofilms are complex structures composed of multilayers of microorganisms, EPS, and water channels [43]. The microorganisms in the biofilms are in different states: metabolically active, metabolically inactive, and dead cells. Therefore, it is important to use more than one technique for biofilm studies. Nowadays, it is difficult to use epifluorescence microscopy as a counting technique for cells in biofilms, because bacteria in a biofilm usually develop layers and residues such as TSB and EY emit strong auto-fluorescence [16,44]. However, it is possible to observe surviving cells after decontamination treatments, even in those treatments where it is not possible to achieve their expression in culture media, either by the detection limit of the technique or by the metabolic state of the bacteria (sub-lethally damaged cells or non-cultivable but metabolically active cells) [42]. SEM enables observing the architecture of the biofilm, without distinguishing living or dead cells [45]. Hence, for biofilm studies, complementary techniques should be used. 

Disinfectant effectiveness on biofilms varies depending on disinfectant characteristics; type of surface; microorganisms in the biofilm; and other factors such as exposure time and temperature [15,41]. Furthermore, interspecies interactions generated within the biofilms have an effect on the dynamics and resistance within the biofilm [38]. Moreover, food residues rich in proteins, lipids, and carbohydrates decrease disinfectant effectiveness, thereby increasing bacteria survival and encouraging cross-contamination due to the increase in bacterial persistence on FCSs [5,38]. Currently, FCS coating, enzymatic disruption, quorum sensing inhibition, biosurfactants, bacteriophages, bacteriocins, essential oils, furanone derivates, high hydrostatic pressure, non-thermal plasma, ultrasound, and photocatalysis have been proposed for biofilm control [9,46]; however, these communities still represent a considerable challenge to food industries and scientists.

## 4. Materials and Methods

### 4.1. Bacterial Strains

The microorganisms used to biofilm formation were *E. coli* ATCC 11303, *S*. Typhimurium ATCC 14028, *S*. Enteritidis ATCC 13076, *P. aeruginosa* ATCC 15442, *L. monocytogenes* ATCC 19111, and *B. cereus* ATCC 14579 (vegetative stage). Before utilization, the microorganisms were incubated individually in TSB (Becton Dickinson Bioxon, Le Pont de Claix, France) at 37 °C for 24 h in aerobic and static conditions to yield a final concentration of 10^7^ CFU/mL.

### 4.2. Chemical and Enzymatic Agents

The assessed products were Sanicip Bio Control (active product obtained of the mixture of SBC 1 and SBC 2, National Sanitation Foundation (NSF) numbers 155919 and 155920, respectively) and Sanicip PAA (peracetic acid, PAA; 200 mg/L, NSF number 144381) (CIP & GROUP, Tlajomulco de Zuñiga, Mexico). Deterzyme 520/180 is a mixture of alkaline protease and α-amylase produced by *Bacillus licheniformis* and *Bacillus subtilis*, respectively (ENMEX, Tlalnepantla, Mexico), which was used to the assessment of biofilm removal. 

### 4.3. Microbicidal Activity against Planktonic Cells

Bactericidal efficacy assays were performed according to AOAC Official Method 960.09 09 [21] with the products SBC (8 mL/L) and Sanicip PAA (200 mg/L). The concentrations used are approved for hard surfaces [23]. Briefly, 100 µL of overnight cultures (1 × 10^7^ c/mL) were mixed by vortexing for 15 s (Vortex Genie 2, Model G-560), with 9.9 mL disinfectant solution with or without 100 g/L of organic matter (meat extract, Becton Dickinson & Co., Le Pont-de-Claix, France; egg yolk or WM processed at ultra-high temperatures purchased from a retail shop in Jalisco, Mexico). After 30 s, 100 µL of the assay mix was transferred to a new Eppendorf tube with 900 mL of Dey/Engley (D/E; Becton, Dickinson and Company, Le Pont de Claix, France) broth to neutralize the disinfectant activity. After 30 min of contact with D/E medium, the number of surviving bacteria was estimated by standard plate counting on tryptic soy agar (TSA; Becton Dickinson, Le Pont de Claix, France) and incubated at 37 °C for 24 h in aerobic conditions. Each assay was performed in triplicate. The percentage of reduction was calculated with the following formula:

$$Reduction\;\left(\%\right)\;=\;100\;-\;\frac{S\left(100\right)}{APC}$$

where *S* = surviving bacteria (CFU/mL) and *ACP* = aerobic counting plate initial (CFU/mL). The disinfectant was considered effective when it demonstrated a 99.999% bacterial reduction.

### 4.4. Biofilm Development

#### 4.4.1. Contact Surfaces

The SS (AISI 304, 2 × 1 × 0.1 cm; CIMA Inoxidables, Guadalajara, Mexico) and PP coupons (2 × 1 × 0.2 cm; Plásticas Tarkus, Tlaquepaque, Mexico) were cleaned. Briefly, the surfaces were immersed in pure acetone (Fermont, Monterrey, Mexico) for 1 h to remove any debris and grease, immersed in neutral detergent (30 mL/L; Cip & Group S. de R.L., Tlajomulco de Zuñiga, Mexico) for 1 h, rinsed with sterile distilled water (SDW), cleaned with ethanol (700 mL/L; Hycel, Zapopan, Mexico), dried for 2 h at 60 °C, and sterilized by autoclaving (121 °C for 15 min) [47].

#### 4.4.2. Biofilm Development and Quantification

Mixed-species biofilms were developed in three culture media: TSB with 100 mL/L chicken egg yolk, (TSB+EY), TSB with 100 g/L meat extract (TSB+ME), and WM. Briefly, each coupon was individually introduced into a new polypropylene tube (15 mL Centrifuge tube, Corning CentriStar, New York, NY, USA) containing 5 mL of the corresponding culture media and was inoculated with 25 µL of each bacterial species (1 × 10^6^ CFU/mL). *S*. Typhimurium was used in biofilms developed in TSB+ME and WM and *S*. Enteritidis was inoculated in TSB+EY. The tubes with the coupons were incubated at 25 °C for 120 h. After this period, the coupons were removed from the tube, immersed into a new fresh medium and inoculated with the same microorganisms (1 × 10^6^ CFU/mL), and incubated for 120 h. At the end of the incubation period, the coupons were removed from the tube using sterile forceps, rinsed by vortexing (150 rpm/10 s) in 5 mL of Dulbecco’s phosphate-buffered saline (PBS; Sigma-Aldrich, St Louis, MO, USA). The conventional plate counting on tryptic soy agar with lactose (10 g/L; Sigma–Aldrich, St. Louis, MO, USA) and phenol red (0.1 g/L; Hycel, Zapopan, Jalisco, Mexico) was realized. For quantification of *E. coli* and *B. cereus* in multispecies biofilms, cefsulodin (50 μg/mL; Sigma–Aldrich, St. Louis, MO, USA) and polymyxin B (70 μg/mL; Sigma–Aldrich, St. Louis, MO, USA) were added to culture media, respectively. Petri dishes were incubated at 37 °C for 24 h. Colonies of *E. coli* and *L. monocytogenes* were yellow due to lactose fermentation; the other microorganism colonies were colorless. *Salmonella* and *P. aeruginosa* were distinguished using the oxidase test [19]. Each quantification was carried out in triplicate. Controls without microorganisms were included for the determination of contamination.

### 4.5. Removal and Disinfection Treatment Procedures

At the end of the incubation period, the coupons were removed from the tubes using sterile forceps and rinsed as above. Then, treatments to remove biofilms with SBC 30 mL/L or with a mixture of alkaline protease and α-amylase produced by *Bacillus licheniformis* and *Bacillus subtilis*, respectively (Deterzyme 520/80; ENMEX, Tlalnepantla, Mexico), were applied according to Figure 1. PAA at 200 mg/L was used as a disinfectant. Treatment with SDW at 30 °C/25 min was incorporated as a control. At the end of time exposure, each coupon was transferred to D/E broth. After 30 min in D/E broth, the surviving cells were estimated by counting plates, as in the Section 4.4.2. Each treatment was evaluated in triplicate.

### 4.6. Microscopy Analysis

#### 4.6.1. Epifluorescent Microscopy

Before and after removal and disinfection treatments, biofilms developed on SS were rinsed with PBS as above, stained with 5(6)-carboxyfluorescein diacetate (CFDA, 10 µg/mL; Sigma Aldrich, St Louis, MO, USA), and dried in a cabinet biosafety level II. The CFDA excess was rinsed with SDW. The coupons were observed under a Nikon Eclipse E400 Epifluorescent Microscope using a 100× oil immersion lens and a BA 515 B-2A filter at 450–490 nm; at least 18 fields were observed [40]. 

#### 4.6.2. Scanning Electron Microscopy (SEM) Analysis

Before and after removal and disinfection treatments, coupons of each material (SS and PP) were rinsed with PBS as above, then was immersed in 20 mL/L glutaraldehyde (DermoDex, Tlalpan, CDMX, Mexico) for 2 h at 4 °C to fix the biofilm. After serial dehydration in ethanol (30, 50, 60, 70, 90, and 95 mL/100 mL) for 10 min each at 4 °C, every coupon was rinsed (three 10 min rinses) in absolute ethanol [48]. Samples were dried and were coated with gold for 30 s [49]. Biofilms were observed under a TESCAN Mira 3 LMU Model field emission scanning electron microscope (Brno-Kohoutovice, Czech Republic).

### 4.7. Statistical Analysis

All of the experiments were performed in triplicate; the statistical analysis was carried out using ANOVA; the percentages data were arcsine square root transformed. The variances were examined by the least significant difference (LDS) test in the software Statgraphics Centurion XVI.I (Statpoint Technologies, Inc., Warrenton, VA, USA).

## 5. Conclusions

The resistance of mixed-species biofilms developed by *E. coli*, *S. typhimurium*, *S. enteritidis*, *P. aeruginosa*, *L. monocytogenes*, and *B. cereus* under conditions that simulate the dairy, meat, and egg processing industries was strongly affected by the type of FCS and surrounding media. The use of Sanicip Bio Control and enzymes plus Sanicip PAA were effective in removing the biofilms developed on SS. Hence, efforts should be conducted to prevent cell aggregation, promote the use of hydrophilic materials such as stainless steel, and use protocols of cleaning and disinfection based on the use of biological and chemical agents. Moreover, enzymatic agents are a great alternative to biofilm control in the food industry, establishing their use according to the type of food residues, potential microorganisms in the biofilm, and optimum temperature to maximize their activity. These results can contribute to applying novel approaches for controlling biofilms in food processing environments, improving food safety and quality.

## Figures and Tables

**Table 1:** Microorganisms recovered from mixed-species biofilms developed on stainless steel AISI 304 before and after removal treatments.

Culture Media	Microorganism	Initial Count ^a^	Treatments ^b^					
			SDW	SBC	SBC + PAA	Enz	Enz + PAA	Enz + SBC + PAA
**TSB + meat extract (100 g/L)**	*E. coli*	4.41 ± 0.17 H^c^a^d^	4.70 ± 0.39 CDa	ND	ND	4.19 ± 0.22 BCDa	ND	ND
	*S.* Typhimurium	6.11 ± 0.13 BCa	5.28 ± 0.42 BCb	ND	ND	4.66 ± 0.28 Bc	ND	ND
	*P. aeruginosa*	6.26 ± 0.27 ABCa	4.83 ± 0.20 BCDb	ND	ND	4.32 ± 0.03 BCc	ND	ND
	*L. monocytogenes*	4.68 ± 0.10 GHa	4.41 ± 0.17 Da	ND	ND	3.43 ± 0.29 Fc	ND	ND
	*B. cereus*	ND	ND	ND	ND	ND	ND	ND
**TSB + egg yolk (100 mL/L)**	*E. coli*	5.61 ± 0.24 DEa	4.30 ± 0.65 Db	ND	ND	3.62 ± 0.54 DEFb	ND	ND
	*S.* Enteritidis	5.84 ± 0.13 CDa	5.28 ± 0.43 BCa	ND	ND	5.35 ± 0.40 Aa	ND	ND
	*P. aeruginosa*	6.52 ± 0.08 Aa	5.30 ± 0.39 BCb	ND	ND	3.86 ± 0.48 CDEFc	ND	ND
	*L. monocytogenes*	4.46 ± 0.28 Ha	4.31 ± 0.39 Dab	ND	ND	3.61 ± 0.38 EFb	ND	ND
	*B. cereus*	1.46 ± 0.28 Ia	ND	ND	ND	ND	ND	ND
**Whole milk**	*E. coli*	5.73 ± 0.38 DEa	5.39 ± 0.28 Ba	ND	ND	4.62 ± 0.32 Bb	ND	ND
	*S.* Typhimurium	6.50 ± 0.29 ABa	6.09 ± 0.28 Aab	ND	ND	5.91 ± 0.24 Ab	ND	ND
	*P. aeruginosa*	5.39 ± 0.21 EFa	4.90 ± 0.24 BCDb	ND	ND	4.09 ± 0.39 BCDEb	ND	ND
	*L. monocytogenes*	4.96 ± 0.38 FGa	5.03 ± 0.58 BCDa	ND	ND	3.49 ± 0.35 EFb	ND	ND
	*B. cereus*	ND	ND	ND	ND	ND	ND	ND

^a^ Mean of three tests of initial population before removal treatments in log_10_ ± standard deviation (*n* = 3); ^b^ mean of three tests of microorganisms recovered after removal treatments in log_10_ ± standard deviation (*n* = 3); ^c^ values in the same column with different capital letter are significantly different (*p* ≤ 0.05); ^d^ values in the same row with different lowercase letter are significantly different (*p* ≤ 0.05).; initial cellular densities 6.46 ± 0.20, 6.67 ± 0.09 and 6.55 ± 0.27 Log_10_ CFU/cm^2^ in TSB+ME, TSB+EY, and WM.; SDW: sterile distilled water; SBC: Sanicip Bio Control (30 g/L, 30 min, 25 °C); PAA: Sanicip PAA (200 mg/L, 10 min, 25 °C); Enz: enzymatic treatment (180 MWU/g, 30 min, 25 °C; MWU: modified Wohlgemuth unit). ND: not detected after removal treatment. Detection limit: 0.81 log_10_ CFU/cm^2^.

**Table 2:** Microorganisms recovered from mixed-species biofilms developed on polypropylene before and after removal treatments.

Culture Media	Microorganism	Initial Count ^a^	Treatments ^b^					
			SDW	SBC	SBC + PAA	Enz	Enz + PAA	Enz + SBC + PAA
TSB + meat extract (100 g/L)	*E. coli*	5.30 ± 0.49 D^c^a^d^	4.87 ± 0.64 Fa	2.70 ± 0.39 DEb	ND	4.88 ± 0.36 DEFa	2.12 ± 0.13 Fc	ND
	*S.* Typhimurium	6.89 ± 0.38 CBa	6.42 ± 0.03 CDb	4.29 ± 0.21 Cc	3.33 ± 0.46 BCd	6.54 ± 0.17 ABb	3.52 ± 0.06 Bd	2.37 ± 0.16 CDe
	*P. aeruginosa*	7.11 ± 0.43 ABCa	5.62 ± 0.18 Eb	4.14 ± 0.63 CDc	2.89 ± 0.29 Cd	5.52 ± 0.31 Cb	2.79 ± 0.29 DEde	2.17 ± 0.41 DEe
	*L*. *monocytogenes*	5.42 ± 0.40 Da	4.93 ± 0.11 Fa	3.69 ± 0.32 BCb	ND	4.90 ± 0.33 DEa	3.01 ± 0.23 CDc	ND
	*B. cereus*	3.56 ± 0.01 Ea	2.08 ± 0.41 Gb	ND	ND	2.89 ± 0.42 Gb	ND	ND
TSB + egg yolk (100 mL/L)	*E. coli*	5.72 ± 0.41 Da	5.12 ± 0.32 Fa	ND	ND	4.47 ± 0.36 EFb	ND	ND
	*S.* Enteritidis	6.97 ± 0.27 ABCa	6.50 ± 0.06 Cb	4.07 ± 0.19 ABc	3.51 ± 0.29 Bd	6.42 ± 0.11 Bb	4.07 ± 0.10 Ac	3.05 ± 0.03 Be
	*P. aeruginosa*	6.63 ± 0.30 Ca	6.02 ± 0.17 DEb	3.48 ± 0.65 CDd	2.28 ± 0.21 Def	5.38 ± 0.17 Cc	2.45 ± 0.27 Eef	2.08 ± 0.25 DEf
	*L*. *monocytogenes*	5.03 ± 0.58 Ea	4.90 ± 0.11 Fa	ND	ND	4.51 ± 0.29 Fb	ND	ND
	*B. cereus*	3.06 ± 0.47 Ea	2.17 ± 0.22 Gb	ND	ND	ND	ND	ND
Whole milk	*E. coli*	7.33 ± 0.22 ABa	7.30 ± 0.45 Ba	2.20 ± 0.45 Ed	3.84 ± 0.40 ABc	6.18 ± 0.05 Bb	2.35 ± 0.21 DEFd	1.64 ± 0.32 Ee
	*S.* Typhimurium	8.11 ± 0.06 Aa	7.64 ± 0.27 Ab	3.13 ± 0.52 CDe	4.29 ± 0.47 Ad	6.92 ± 0.37 Ac	3.22 ± 0.15 Ce	3.10 ± 0.08 Ae
	*P. aeruginosa*	6.43 ± 0.17 Ca	5.62 ± 0.35 Eb	2.93 ± 0.23 CDd	3.73 ± 0.06 Bc	5.53 ± 0.27 Cb	2.84 ± 0.07 Dd	2.27 ± 0.11 BCd
	*L*. *monocytogenes*	5.70 ± 0.15 Da	5.20 ± 0.52 Eab	2.49 ± 0.11 DEc	ND	5.25 ± 0.03 CDb	1.94 ± 0.24 Gd	ND
	*B. cereus*	ND	ND	ND	ND	ND	ND	ND

^a^ Mean of three tests of initial population before removal treatments in log_10_ ± standard deviation (*n* = 3); ^b^ mean of three tests of microorganisms recovered after removal treatments in log10 ± standard deviation (*n* = 3); ^c^ values in the same column with different capital letter are significantly different (*p* ≤ 0.05); ^d^ values in the same row with different lowercase letter are significantly different (*p* ≤ 0.05); initial cellular densities 7.18 ± 0.54, 7.07 ± 0.28, and 7.91 ± 0.07 log10 CFU/cm2 in TSB+ME, TSB+EY and WM; SDW: sterile distilled water; SBC: Sanicip Bio Control (30 g/L, 30 min, 25 °C); PAA: Sanicip PAA (200 mg/L, 10 min, 25 °C); Enz: enzymatic treatment (180 MWU/g, 30 min, 25 °C; MWU: modified Wohlgemuth unit); ND: not detected after removal treatment. Detection limit: 0.71 log10 CFU/cm^2^.

## Data Availability

The data used to support the findings of this study are available from the corresponding authors upon request.
